# Influence of Drought Stress on Physiological Responses and Bioactive Compounds in Chicory (*Cichorium intybus* L.): Opportunity for a Sustainable Agriculture

**DOI:** 10.3390/foods11223725

**Published:** 2022-11-20

**Authors:** Sebastiano Delfine, Alessandra Fratianni, Annacristina D’Agostino, Gianfranco Panfili

**Affiliations:** Dipartimento di Agricoltura, Ambiente e Alimenti, Università degli Studi del Molise, Via De Sanctis, 86100 Campobasso, Italy

**Keywords:** drought stress, chicory, tocopherols, carotenoids, sustainable agriculture, yield

## Abstract

Food production from agriculture depends on irrigation, mainly in poor rainfall zones, such as the Mediterranean region. Chicory is an important food crop component of the Mediterranean diet. Considering the increasing incidence of drought due to climate change, this study was carried out in order to investigate the effect of moderate drought stress on photosynthesis, leaf gaseous exchange, growth, and tocol and carotenoid composition of chicory under field conditions. Chicory was subjected to rainfed condition stress in a randomized block design. At 50 days of treatment, drought stress caused about 48% reduction in dry matter, 30% in leaf relative water content, and about 25% in photosynthetic rate and stomatal conductance, whereas mesophyll conductance was not affected. A strong relationship between photosynthetic rates and stomatal conductance was observed. In the rainfed chicory, at the end of treatment, an increase (about 20%) in carotenoid and tocopherol content was found, thus, giving further insight into the positive effect of moderate drought stress on these compounds. This finding suggests that under proper rainfed conditions, it is possible to increase and save the quality of dry chicory, although yield loss occurs.

## 1. Introduction

Chicory (*Cichorium intybus* L.) is a native plant of the Asteraceae family, common in the Mediterranean basin and belonging to the so-called Mediterranean diet [1]. It is a widely spread crop able to tolerate different climatic and soil conditions, and is commercially cultivated in North America, Europe, and some Asian regions [2]. Several studies have reported its health benefits [3,4,5].

Agricultural productivity is strongly dependent on water availability, mainly in arid and semi-arid zones [6]. In the last years, climatic change caused a rainfall decrease in different areas of the Mediterranean countries; consequently, a severe deficit in the available water resources for agricultural production occurred [7]. The irregular distribution of rainfall became the main limitation to a sustainable crop yield in the drought-prone areas [8]. Strong water stress limits crop yields and growth, leading to negative economic consequences [9]. To reduce the negative effects of drought, crops require enough water availability during summer in order to guarantee growth and high yields. Different papers report the positive effect of water availability on yield in chicory and the strategies for mitigating water-deficit stress [10,11,12,13]. The reduced water availability imposes changes to chicory plant morphological traits, such as canopy structure, and negatively affects biomass accumulation, as in mallow plants [14,15]. Plants may develop different morpho-physiological and adaptation mechanisms in response to environmental stresses [16]. An increase in the antioxidant systems, such as enzymes and secondary metabolites (ascorbic acid, glutathione, tocols, carotenoids, flavones, and flavonoids) has been observed to enhance drought tolerance [17,18,19], even if this behavior was found to depend on phenological stage, genotype, organs of the plants (fruits and leaves), severity and length of the stress [20,21]. In some studies on fruits and vegetables under water stress conditions, an increment of β-carotene and carotenoids was found [22,23,24], while, in other studies, a reduced tissue concentration of carotenoids was observed [25,26]. 

Carotenoids (carotenes and xanthophylls) are yellow, orange, and red pigments biosynthesized by photosynthetic organisms, present in various fruits and vegetables. Xanthophylls, in particular, can be found in their free oxygenated form or esterified to fatty acids. Carotenes have an important function in the diet for their provitamin A, antioxidant and immunosystem activity, and for intercellular communication. Carotenoids cause a significant reduction in the risk for different diseases [27]. In plants, besides their direct role in photosynthesis, they are involved in the mechanisms of oxidative stress tolerance [28,29].

An increase in α-tocopherol was observed in response to water stress [30,31,32]. Alfa-tocopherol is included in a group of vitamers, β, γ, δ- tocopherol (T), and α, β, γ,- tocotrienol (T3), known as Vitamin E. Tocopherols are involved in plant growth, signal transduction, phytohormonal balance, abscission, and senescence, as well as in many other metabolic processes [30,31,33]. They are also known to physically quench and chemically react with O_2_ in chloroplasts, thus, protecting lipids, other membrane components, and the structure and functions of PSI [30,32]. As the major lipid soluble chain-breaking antioxidants in humans, they have been demonstrated to prevent different chronic diseases [34]. Vegetable oils are their main sources [35], but they are present to a different extent in several vegetable products at significant nutritional amounts. 

The aim of this study was to investigate how moderate drought stress, through rainfed conditions, can affect growth parameters, including the physiological, agronomical, and nutritional traits of field-grown chicory. This in order to verify if it could be an effective agricultural practice to be adopted for chicory in order to increase the quality of plants and to maintain a sustainable crop productivity in the southern Italy Mediterranean environment. 

## 2. Materials and Methods

### 2.1. Cultural Practices and Experimental Treatments

Field trials were carried out during 2020 and 2021, on a chicory plant (cv. Choice), at an experimental field site in Baranello (Molise Region, Italy, latitude 41°31′ N, longitude 14°33′ E, altitude 630 m a.s.l.). The experimental field soil had a uniform profile, with an organic matter content of 1.5% and a clay-sand texture. It contained 0.12% of total N (nitrogen), 20.5 µg/g of available P (phosphorous), 139 µg/g of exchangeable K (potassium), and very low active CaCO_3_. The pH was, on average, neutral and the salinity was low. The previous crop was *Phaseolus vulgaris* L. Moderate drought was imposed through rainfed conditions for 50 days. Rainfed (R) and well-watered (W) plants were compared following a randomized block design with five replications (3 m^2^ each plot). Sowing was carried out manually, placing seeds at a 0.5 cm depth and spacing the rows at 45 cm. Thinning was made to a plant population of 100 plants/m^2^. After sowing, in order to ensure a uniform crop establishment, the same irrigation amount was applied to all fields, by applying a drip irrigation system on every row. Whenever evapotranspiration (ET) reached 25 mm, water restorations occurred. The Penman–Monteith formula was used to calculate ET, from micrometeorological data [16]. At the beginning of the second growing season (25 April), the plants were cut to 30 mm above the potting media level and rainfed conditions were imposed. The first defoliation occurred on the 24 May 2021, 30 days after treatment (30 DAT), when plants showed at least seven fully developed leaves, and was made manually. The second defoliation occurred on the 15 June 2021 (50 DAT), in the same manner as the first. All plots were treated with the recommended fertilizer rates of the area, 70 kg/ha of P_2_O_5_ and 80 kg/ha of K_2_O at seedbed preparation and 100 kg/ha of N applied in two splits. The first split (60% of total N rate) was basally added to the soil the sowing day, while the second split (40% of total N rate) was supplied at the beginning of the next growing season. To allow for uniform growing conditions, a buffer strip surrounded the field. The weather data (temperature and rainfall) were taken from a meteorological station situated near the experimental field (Table 1).

### 2.2. Leaf Traits and Gas-Exchange Measurements

Between May and June of the second year (at 30, 37, 43, and 50 DAT), eight leaf gas-exchange measurements were performed, before the flowering stage, using a portable infrared gas analyzer (Li-6400; LI-COR, Lincoln, NE, USA). Leaf photosynthetic capacity (Pn), stomatal (gs), and mesophyll conductance (gm) were calculated, as in Delfine, Loreto, Pinelli, Tognetti, and Alvito [36]. The leaf gas exchange data were measured to the fully expanded leaves until 11.30 a.m., in order to avoid the midday depression in photosynthetic rate. The relative water content (RWC), i.e., the ratio of water content in fresh to turgid leaves, was also measured on the same leaves used for gas-exchange measurements [10]. 

### 2.3. Chemicals and Reagents

Solvents were obtained at the highest purity; other reagents were of analytical grade (Sigma Chemicals, St. Louis, MO, USA). Violaxanthin, neoxanthin α-carotene, 9-*cis*-β-carotene, and 13-*cis*-β-carotene standards were obtained from CaroteNature (Lupsingen, Switzerland); lutein, zeaxanthin, and β-cryptoxanthin were purchased from Extrasynthese (Z.I. Lyon-Nord, Genay, France). All-*trans*-β-carotene was from Sigma Chemicals; α, β, γ, and δ-tocopherol standards were from Merck (Darmstadt, Germany); α, β, γ, and δ-tocotrienol standards were obtained as in [37]. 

### 2.4. Nutritional Analysis

Full-expanded leaves from W and R samples were harvested at 30 DAT and 50 DAT. The non-edible part was removed. From each sample, a minimum of 500 g was collected and cleaned by removing foreign parts. Then, samples were freeze-dried (Genesis 25SES freeze dryer, VirTis Co., Gardiner, NY, USA) and ground using a refrigerated IKA A10 laboratory mill (Staufen, Germany), then mixed and stored at −20 °C. The AOAC methods were used to determine moisture, proteins, ash, and fiber [38]; fats were analyzed by the method of acid hydrolysis [39]. Tocols and carotenoids were extracted according to Fratianni et al. [3] and determined according to Panfili, Fratianni, and Irano [37,40].

Carotenoids were analyzed through a normal (for xanthophylls) and a reverse phase (for carotenes) HPLC method, as in [3,40]. Analysis were performed by using a HPLC Dionex (Sunnyvale, CA, USA) analytical system, comprising a 50 μL injector loop (Rheodyne, Cotati) and a U6000 pump system. Tocol determination was carried out by a Dionex HPLC, through normal phase, as in [3,37]. All tocols were fluorimetric detected by means of a Dionex RF 2000 spectrofluorimeter, at an excitation wavelength of 290 nm and an emission wavelength of 330 nm. Compounds were identified through their spectral characteristics and by comparison of their retention times with standard solutions. Calibration curves of each standard solution were used for quantification. 

### 2.5. Statistical Analysis

Agronomical (fresh and dry biomass) and physiological data (RWC, photosynthesis, stomatal conductance, and mesophyll conductance) were the means of five replicates. Results of nutritional composition and bioactive compounds were the average of three determinations. Data were analyzed by means of the ANOVA test, using a Statistical Software Package for Windows (SPSS Inc., Chicago, IL, USA). The significance of difference was set at *p* ≤ 0.05. 

## 3. Results and Discussion

### 3.1. Weather Data

The rainfall distribution was that of the period and varied between before and throughout the trial. It was higher from January to April (maximum 102.6 mm) and almost completely absent throughout the experimental trial (0.4 mm). Air temperature was that of the period, with a maximum average temperature of about 30 °C (Table 1).

### 3.2. Fresh Biomass and Dry Matter Accumulation

**Figure 1:**
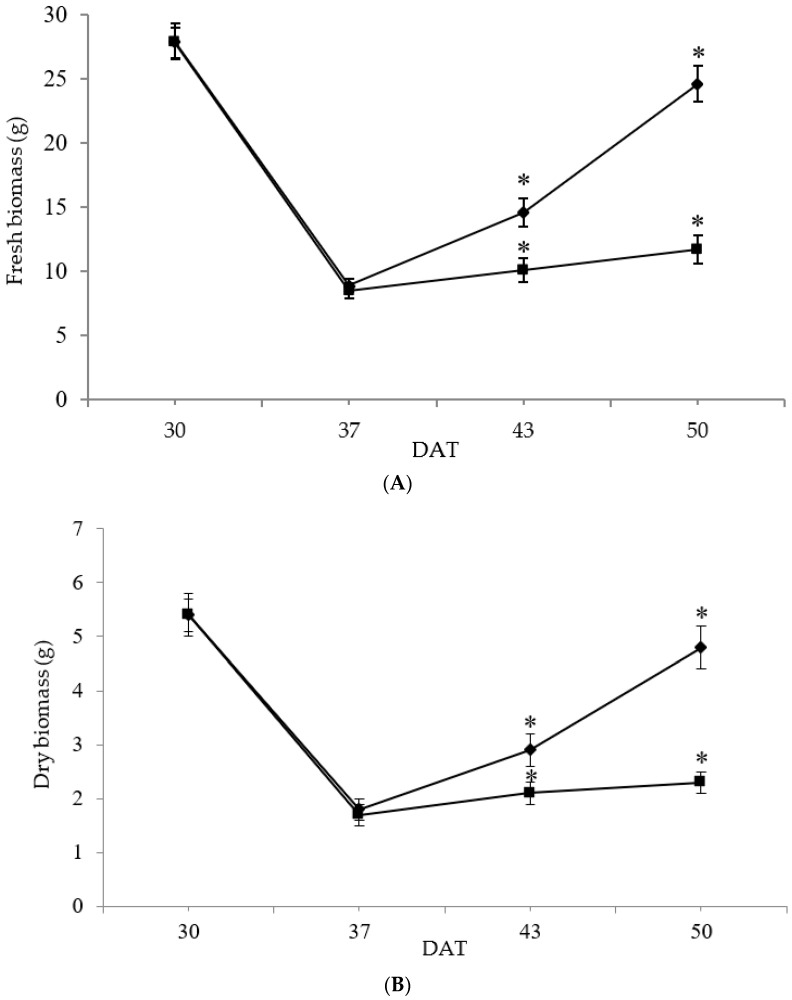
Changes in fresh biomass (g) (**A**) and in dry biomass (g) (**B**) of chicory exposed to the following treatments: rainfed (■) and well-watered (•). Measurements were performed at 30, 37, 43, and 50 days after treatment (DAT). Values are expressed as mean ± standard deviation (*n* = 5). Asterisks indicate significant differences between treatments on the given DAT at *p* ≤ 0.05.

The aboveground fresh biomass accumulation (g) was significantly affected by drought stress (Figure 1A). The R treatment resulted in a reduction in chicory biomass of 30% and 52%, compared to W, at 43 and 50 DAT, respectively. Results also showed that drought stress significantly affected fresh biomass accumulation at 50 DAT, compared to the same treatment at 30 DAT. At 30 DAT, no significant difference between treatments was observed, while, at 50 DAT, rainfed plants showed a 48% decrease in dry matter compared to the well-watered ones, (Figure 1B), The decline in dry matter suggests that the decrease in photosynthesis resulted in a reduction in translocation of photo-assimilates to plant tissues, leading to smaller plants [16]. The literature data indicate a reduction in dry matter due to drought stress in chicory [11,12,13,41].

### 3.3. Relative Water Content

**Figure 2:**
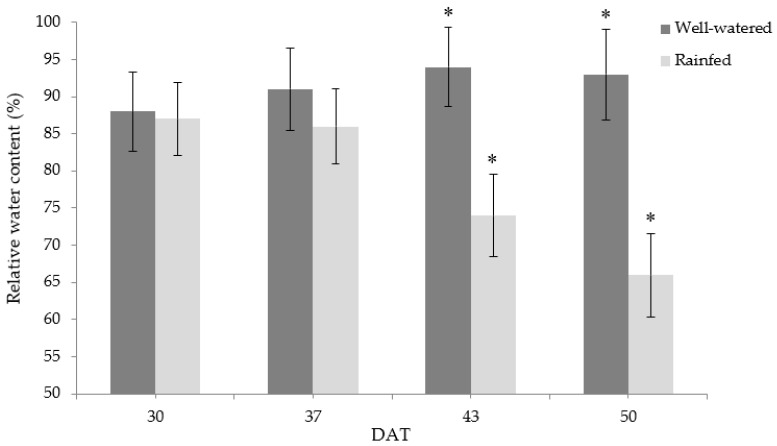
Changes in relative water content (%) of chicory subjected to the following treatments: rainfed (R) and well-watered (W). Measurements were performed at 30, 37, 43, and 50 days after treatment (DAT). Bars represent mean ± standard deviation (*n* = 5). Asterisks indicate significant differences between treatments on the given DAT at *p* ≤ 0.05.

Rainfed conditions caused a reduction in relative water content (RWC) in chicory leaves (Figure 2). At 30 and 37 DAT, no significant difference between treatments was observed, which may be attributed to the similar tissue water content. At 43 DAT, the relative water content of R leaves was significantly lower than that of W. At 50 DAT, R leaves resulted in the lowest RWC, with a 29% reduction compared to W. The leaf RWC was closely associated with photosynthetic gas exchange parameters during different crop growth stages.

### 3.4. Photosynthesis, Stomatal (gs) and Mesophyll (gm) Conductance

**Figure 3:**
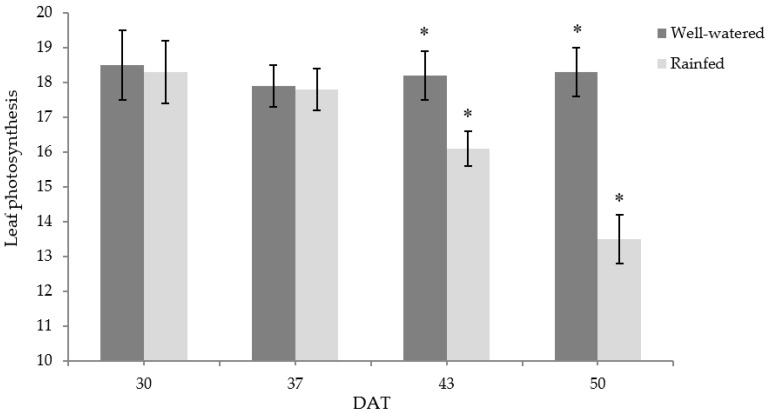
Changes in photosynthesis (µmol m^−2^ s^−1^) of chicory subjected to the following treatments: rainfed (R) and well-watered (W). Measurements were performed at 30, 37, 43, and 50 days after treatment (DAT). Bars represent the mean ± standard deviation (*n* = 5). Asterisks indicate significant differences between treatments on the given DAT at *p* ≤ 0.05.

**Figure 4:**
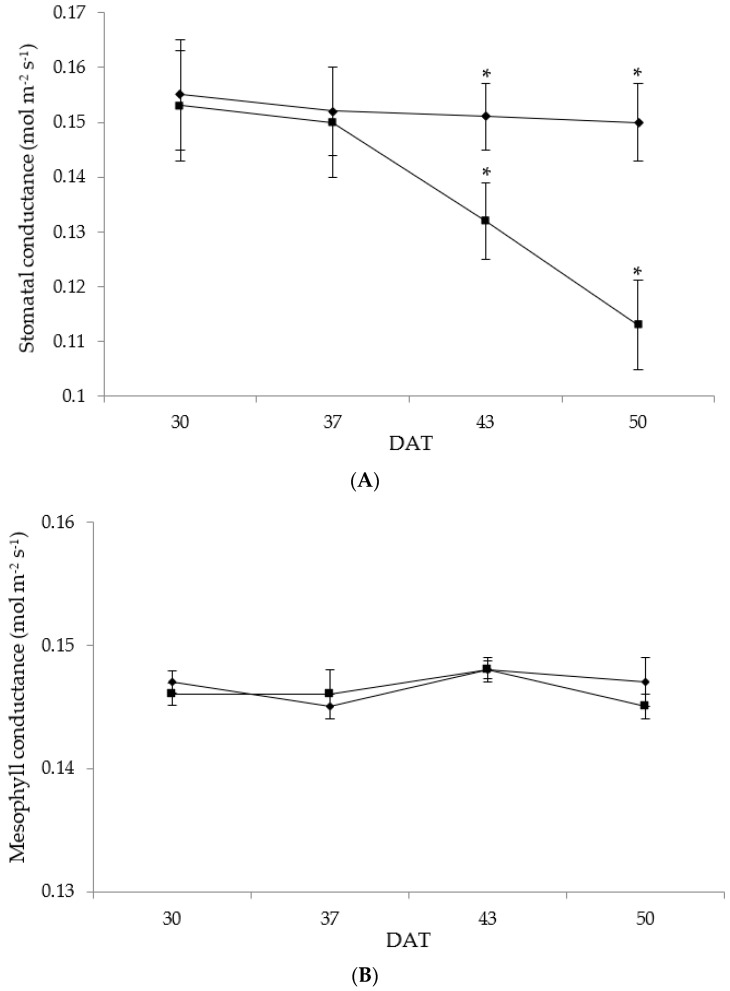
Changes in stomatal conductance (mol m^−2^ s^−1^) (**A**) and in mesophyll conductance (mol m^−2^ s^−1^) (**B**) of chicory subjected to the following treatments: rainfed (■) and well-watered (•). Measurements were performed at 30, 37, 43, and 50 days after treatment (DAT). Values are expressed as mean ± standard deviation (*n* = 5). Asterisks indicate significant differences between treatments on the given DAT at *p* ≤ 0.05.

Rainfed conditions caused a significant effect on photosynthesis (Pn) at the end of the trial period (50 DAT) (Figure 3), with the highest photosynthetic rates in W and the lowest in R. These results suggest that, during the growth stage of rainfed samples, drought-stressed chicory can experience a serious reduction in the photosynthetic rates that can be higher than 25%. The reduction in photosynthetic rates can depend on stomatal and non-stomatal factors [16]. The highest stomatal conductance (gs) was found when the plant was at the first defoliation (30 DAT), while it reduces, only under rainfed conditions, as the plant grows to the second defoliation at 50 DAT (Figure 4A). In particular, at 30 DAT, a similar value of gs was found between R and W, while, at 43 and 50 DAT, it was significantly affected by rainfed conditions, showing, at the end of treatment, a 24% reduction in R plants. At 37 DAT, a very strong relationship (r^2^ = 0.989) between photosynthetic rates and stomatal conductance was observed. A reduction in the photosynthetic rate during drought stress, due to stomatal closure, has been reported in chicory [12,13], as well as in pepper, mint, and rosemary [16,36]. The results found revealed that, throughout the trial, the mesophyll conductance (gm) of R plants was not significantly different to W (Figure 4B). The weak relationship (r^2^ = 0.117) between the mesophyll conductance and the stomatal conductance suggested that the reduction in stomatal conductance of R plants did not affect mesophyll conductance. Similarly, throughout the trial, there was a weak relationship (r^2^ = 0.192) between mesophyll conductance and photosynthesis. The reduction in photosynthesis observed during the end of the trial could be due to stomatal closure; in this case, a consequent reduction in mesophyll CO_2_ concentration should be observed [42]. The mesophyll components generally cause an additional resistance to CO_2_ diffusion toward the chloroplasts that may increase under stress conditions [16,36,42,43], and it is likely to be controlled by the mesophyll structure [44]. The similar gm observed in irrigated and rainfed leaves, during the whole harvest time, indicated that gm did not contribute to increasing the resistance to CO_2_ diffusion in rainfed chicory leaves. This result revealed that photosynthesis was not directly affected by the resistances to CO_2_ diffusion. The reduction in stomatal conductance might have resulted from stomatal closure, which prevents CO_2_ from entering the leaf, leading to a decrease in photosynthetic carbon assimilation. The strong relationship between Pn and gs indicated that stomatal closure mostly regulated the reduction in Pn, whereas the weak relationship between Pn and gm demonstrated that the reduction in Pn was not affected by an additional resistance to CO_2_ diffusion toward the chloroplasts [16]. As previously discussed, rainfed conditions restrict chicory plant growth and productivity, also reducing the uptake and the diffusion of CO_2_, and such conditions also alter different biochemical reactions, which further inhibit photosynthesis [16,45].

### 3.5. Nutritional Composition

In Table 2 the chemical composition of R and W samples, at 30 and 50 DAT, is reported. Data are expressed as g 100/g dry weight (d.w.). Results are in accordance with the values in the literature [5,46]. A slight significant decrease in fats and increase in proteins was found between the two sampling times. Between the two water regimes, no significant differences were observed.

Eight carotenoid compounds were detected and identified, as follows: violaxanthin, neoxanthin, lutein, zeaxanthin (xanthophylls), and α-carotene, β-carotene, 9-*cis*-β-carotene, and 13-*cis*-β-carotene (carotenes) (Table 3). Lutein was the main carotenoid (about 55–100 mg/100 g d.w.), while β-carotene accounted for 13–20 mg/100 g d.w. Quite high amounts of violaxanthin and neoxanthin were also found. Lutein and β-carotene have been reported as being among the major carotenoids found in green leafy vegetables, even if also other carotenes and xanthophylls were detected [3,4,47]. Results are in the same order of magnitude of different data in the literature [3,4,48]. At 50 DAT in R samples, a significant increment of xanthophylls was observed, ranging from about 27% for neoxanthin to 44% for zeaxanthin. No significant increases of carotenes were found. The total carotenoid amount was, significantly, 22% higher in rainfed samples. 

In accordance with other papers in the literature [3,49], only α-tocopherol (α-T), from about 30 mg/100 g d.w. to 40 mg/100 g d.w., and γ-tocopherol (γ-T), from about 10 mg 100/g d.w. to 18 mg/100 g d.w., were detected. No tocotrienols were found. At 50 DAT, as compared to W samples, there was a significant 20% increase in total tocols in R plants, changing from about 15% for α-T to 40% for γ-T (Table 4). 

The down-regulation of photosynthesis can be linked to damage of the photosynthetic apparatus and an increased thermal dissipation, as a photo-protective process. Here, ROS are produced, as a result of over-photooxidation, which disrupts photosynthetic activities by lipid peroxidation. Alfa tocopherol is reported to be involved in response to water deficiency [30,31,32,33]. This occurs in two phases, as follows: in the first phase, α-T is synthetized to scavenge the ROS; in the second phase, tocopherol degradation is induced by a severe stress. The first phase is prevalent in stress-resistant species and, therefore, a higher tolerance to drought resulted in higher α-tocopherol content [31]. According to our results, fruits from water-stressed plants were also found to contain more γ-tocopherol than those from control plants [23]. The induced concentrations of tocopherols depend upon severity of prevailing stress, its intensity, and species-specific response [20,21]. From the literature, there are controversial results on the effect of water stress on carotenoids. From our results, an increase in xanthophylls was observed, similar to the literature papers where an increment in the xanthophyll cycle components was demonstrated in different plant species under stress conditions [25,50,51]. This cycle consists of the enzymatic interconversions of violaxanthin, antheraxanthin, and zeaxanthin in the thylakoid membrane. It can be considered as another important protective mechanism that helps to minimize irreversible oxidative damage to the photosynthetic apparatus. The physiological function of lutein in stress tolerance is not well understood, but different papers report an increase under drought, due to its effect as a secondary barrier [52,53].

## 4. Conclusions

Information coming from our results confirmed the adverse effect of the absence of rainfall, and the consequent drought stress conditions, on the agronomical and physiological parameters of chicory plants. On the contrary, an increase in tocol and xanthophyll amounts after water stress imposition was observed. The latter results add information to the scarce available literature on chicory and give further insights to the evidence that moderate drought stress can positively affect the content of these components. The overall findings indicate that drought stress can be practiced on chicory in areas where a challenge to irrigation water occurs, with important implications for agricultural practices aimed at saving water in arid and semi-arid growing regions.

## Figures and Tables

**Table 1:** Monthly accumulated precipitation, and maximum and minimum average air temperature before and throughout the experimentation.

Month	Average Max Temperature (°C)	Average Min Temperature (°C)	Rainfall (mm)
January	14.9	−2.9	102.6
February	20.9	−4.6	22.2
March	20.1	−1.4	17.2
April	26.8	−0.7	10.6
May	28.7	5.8	0.4
June	29.9	7.9	0.0

**Table 2:** Chemical composition of chicory at different sampling times and water regimes (g/100 g d.w.) *^a^*.

DAT	Samples	Protein	Fat	Ash	Carbohydrates *^b^*
30	W	19.5 (0.1)	1.8 (0.1)	14.5 (0.6)	64.2 (0.1)
	R	18.8 (0.1)	1.8 (0.1)	15.1 (0.1)	64.3 (0.1)
50	W	16.1 (0.4)	2.4 (0.1)	15.2 (0.4)	66.3 (0.9)
	R	15.8 (1.2)	2.7 (0.7)	15.1 (0.1)	66.4 (1.8)

*^a^* All values are reported as mean ± standard deviation (*n* = 3). Abbreviations are as follows: DAT, days after treatment; W, well-watered; R, rainfed. *^b^* calculated by difference.

**Table 3:** Carotenoid content of chicory at different days after water treatment (mg 100/g d.w.) *^a^*.

DAT	Samples	Violaxanthin	Neoxanthin	Lutein	Zeaxanthin	α-Carotene	13-*Cis*-β-carotene	β-Carotene	9-*Cis*-β-carotene	Totals
30	W	10.7 (0.8)	9.9 (1.0)	57.2 (5.5)	5.2 (1.2)	2.6 (0.4)	2.8 (0.4)	13.3 (3.5)	2.4 (0.3)	104.1 (10.6)
	R	11.3 (2.2)	10.5 (1.3)	53.8 (2.1)	5.5 (0.1)	2.4 (0.8)	2.7 (0.5)	13.3 (1.6)	2.1 (0.4)	101.7 (2.5)
50	W	11.3 (1.5) *	12.6 (0.4) *	72.3 (9.3) *	4.1 (0.7) *	2.7 (0.1)	3.4 (0.3)	18.9 (0.9)	3.0 (0.3)	128.4 (0.7) *
	R	15.4 (0.5) *	16.0 (1.1) *	96.4 (4.6) *	5.9 (0.5) *	2.4 (0.6)	3.0 (0.2)	15.1 (2.4)	2.5 (0.3)	156.7 (2.5) *

*^a^* All values are shown as mean ± standard deviation (*n* = 3). Abbreviations are as follows: DAT, days after treatment; W, well-watered; R, rainfed. Asterisks indicate statistically significant difference at *p* ≤ 0.05, at the same DAT.

**Table 4:** Tocopherol content of chicory at different days after water treatment (mg/100 g d.w.) *^a^*.

DAT	Samples	α-T	γ-T	Totals
30	W	30.9 (2.6)	17.6 (1.1)	48.5 (3.7)
	R	31.9 (0.4)	17.5 (0.2)	49.4 (0.5)
50	W	33.4 (1.7) *	11.4 (0.7) *	44.9 (2.4) *
	R	38.5 (0.1) *	15.8 (0.6) *	54.3 (0.5) *

*^a^* All values are shown as mean ± standard deviation (*n* = 3). Abbreviations are as follows: DAT, days after treatment; W, well-watered; R, rainfed. Asterisks indicate statistically significant difference at *p* ≤ 0.05, at the same DAT.

## Data Availability

The data are available from the corresponding author.
